# A bibliometric analysis of toxicology research productivity in Middle Eastern Arab countries during a 10-year period (2003–2012)

**DOI:** 10.1186/1478-4505-12-4

**Published:** 2014-01-21

**Authors:** Sa’ed H Zyoud, Samah W Al-Jabi, Waleed M Sweileh, Rahmat Awang

**Affiliations:** 1 Poison Control and Drug Information Center (PCDIC), College of Medicine and Health Sciences, An-Najah National University, Nablus 44839, Palestine; 2 Department of Pharmacology and Toxicology, College of Medicine and Health Sciences, An-Najah National University, Nablus 44839, Palestine; 3 WHO Collaborating Centre for Drug Information, National Poison Centre, Universiti Sains Malaysia (USM), Penang 11800, Malaysia; 4 Department of Clinical and Community Pharmacy, College of Medicine and Health Sciences, An-Najah National University, Nablus 44839, Palestine

**Keywords:** Bibliometric, Impact factor, Middle Eastern Arab, Scopus, Toxicology

## Abstract

**Background:**

Bibliometric studies are increasingly being used for research assessment by involving the application of statistical methods to scientific publications to obtain the bibliographics for each country. The main objective of this study was to analyse the research productivity originating from 13 Middle Eastern Arab (MEA) countries with articles published in toxicology journals.

**Methods:**

Data from January 1, 2003 till December 31, 2012 were searched for documents with specific words in the toxicology field as a “source title” in any one of the 13 MEA countries. Research productivity was evaluated based on a methodology developed and used in other bibliometric studies. Research productivity was adjusted to the national population and nominal gross domestic product (GDP) per capita.

**Results:**

Documents (n = 1,240) were retrieved from 73 international peer-reviewed toxicology journals. The *h*-index of the retrieved documents was 39. Of the 73 journal titles, 52 (69.9%) have their IF listed in the ISI Journal Citation Reports 2012; 198 documents (16.0%) were published in journals that had no official IF. After adjusting for economy and population power, Egypt (193.6), Palestine (18.1), Kingdom of Saudi Arabia (KSA) (13.0), and Jordan (11.5) had the highest research productivity. Countries with large economies, such as the Kuwait, United Arab Emirates (UAE), and Oman, tended to rank relatively low after adjustment of GDP. The total number of citations at the time of data analysis (August 4, 2013) was 10,991, with a median (interquartile range) of 4 (1–11). MEA collaborated more with countries in the MEA regions (16.7%), especially KSA, Egypt, and UAE, followed by Europe (14.4%), especially with the United Kingdom and Germany.

**Conclusions:**

The present data show a promising rise and a good start for toxicology research activity in toxicology journals in the Arab world. Research output is low in some countries, which can be improved by investing in more international and national collaborative research projects in the field of toxicology.

## Background

Bibliometric studies are increasingly being used for research assessment [1] by involving the application of statistical methods to scientific publications to obtain the bibliographics for each country. These methods are mainly quantitative but are also used to make pronouncements about qualitative pictures of scientific activities [1,2]. Scientific progress is one of the most important indicators for community and economic development of different countries [3]. Well-known databases, such as PubMed, Scopus, Web of Science (i.e., Thomson Reuters Institute for Scientific Information [ISI]), and Google Scholar index international publications in biomedical sciences [4].

Recently, several studies have measured and analysed the outcome of scientific output from Arab countries in different specialities [2,5-10]. In contrast, the evolution of scientific output in the field of toxicology has been poorly explored to date and there are few internationally published reports on research activity in toxicology [11-16]. To the best of our knowledge, there is a lack of data concerning the evaluation of research productivity in toxicology originating from the Arab world.

Thus, estimates of Arab productivity of ongoing research in the field of toxicology may be of interest. In this bibliometric analysis, we sought to evaluate the contribution of different Middle Eastern Arab (MEA) countries in the scientific research field as published in toxicology journals, and as represented by the quantity and quality of published papers. Bibliometric analysis is a useful tool to obtain information about the current state of research in particular areas and allows researchers to identify and undertake new lines of research [17]. This type of analysis is a research method used in library and information sciences and utilises quantitative analysis and statistics to obtain the bibliographical works within a given field, topic, institute, or country [18-20]. The most important bibliometric indicators for research capacity and productivity include the number of publications, the amount of peer-reviewed scientific journal articles, the number of total citations, and the type of publications [20-22]. Such a study will lead to a better understanding of the current and future status of toxicology in the Middle East. Furthermore, evaluation of toxicological research output in the Arab world is important for monitoring and improving this activity; this could help in putting research activities in the Arab countries into perspective.

## Methods

This study obtained data from Scopus published from January 1, 2003 till December 31, 2012. It is assumed that the last decade would project a better picture of the pattern of publications and the citations received. A comprehensive online search was performed using SciVerse, Scopus, which is one of the world’s largest abstract and citation databases of peer-reviewed literature. Scopus contains 41 million records and covers nearly 18,000 titles from 5,000 publishers worldwide, and provides 100% MEDLINE coverage [23]. The Scopus database was developed by Elsevier and combines the characteristics of both Web of Science and PubMed. These characteristics allow for enhanced service for educational and academic needs, medical literature research, and bibliometric analysis. Scopus offers a basic search or an advanced search options. In the basic search, the results for the chosen keywords can be limited by the date of publication, by addition to Scopus, by subject area, and by document type [24]. The search output from Scopus can be presented as a list of 20 to 200 items per page, and extracted documents can be exported to Microsoft Office Excel®. The results can be refined by document type, author name, source title, publications per year, and/or subject area, and a new search can be initiated within the results [24].

The key words entered in Scopus to accomplish the objective of this study were “Toxicology”, “Toxicological”, and “Toxic”, “toxicon”, “toxin”, “toxins”, “ecotoxicology”, “nanotoxicology”, and “neurotoxicology” as Source Title. Then, all 13 Arab countries in the Middle East were entered as country affiliation (i.e., Egypt, Syrian Arab Republic [SAR], Lebanon, Jordan, Iraq, Kingdom of Saudi Arabia [KSA], Kuwait, Bahrain, State of Palestine, United Arab Emirates [UAE], Yemen, Oman, and Qatar). The subject areas selected for this research were: health sciences, life sciences, social sciences, and physical sciences. The resultant search was as follows: your query: (SRCTITLE(toxicology) OR SRCTITLE(toxicological) OR SRCTITLE(toxic) OR SRCTITLE(toxicon) OR SRCTITLE(toxin) OR SRCTITLE(toxins) OR SRCTITLE(ecotoxicology) OR SRCTITLE(nanotoxicology) OR SRCTITLE(neurotoxicology) AND AFFILCOUNTRY(Jordan) OR AFFILCOUNTRY(Egypt) OR AFFILCOUNTRY(United Arab Emirates) OR AFFILCOUNTRY(Saudi Arabia) OR AFFILCOUNTRY(Palestine) OR AFFILCOUNTRY(Bahrain) OR AFFILCOUNTRY(Yemen) OR AFFILCOUNTRY(Syrian) OR AFFILCOUNTRY(Iraq) OR AFFILCOUNTRY(Kuwait) OR AFFILCOUNTRY(Oman) OR AFFILCOUNTRY(Lebanon) OR AFFILCOUNTRY(Qatar)) AND PUBYEAR >2002 AND PUBYEAR <2013. We excluded documents published as errata.

The collected data were used to generate the following information: (a) total and trends of contributions in toxicology fields between 2003 and 2012; (b) MEA authorship pattern and productivity; (c) collaboration patterns; (d) journals in which MEA researchers publish; (e) the classification of journals as ISI or non-ISI; (f) impact factors (IFs) of all publications; (g) number of citations received by the publications; and (h) areas of interest for published papers.

### Ethical approval

Institutional Review Board (IRB) approval exemptions were obtained by authors from An-Najah National University. The IRB considered waiving the requirement to get approval exemptions for protocols that were clearly below minimal risk, and the current research did not involve any interactions with human participants and used a secondary data set.

### Statistical analysis

Data from Scopus were exported to Excel and then to the Statistical Package for Social Sciences (SPSS; SPSS Inc., Chicago, IL, USA) program version 15 for analysis. Continuous data are presented as mean ± standard deviation (SD), and categorical data are expressed as numbers with percentages. Variables that are not normally distributed are expressed as median (Q1–Q3: interquartile range). The *h*-index for the data collected from SCOPUS is presented. The *h*-index represents the number of citations received for each of the articles in descending order and the *h*-graph measures the impact of a set of documents and displays the number of citations per article. The journal’s IF was evaluated using the Journal Citation Report (JCR; Web of Knowledge) 2012 science edition by Thomson Reuters (New York, NY, USA). Publication activity was adjusted for the 13 MEA countries categorized by population size and gross domestic product (GDP) retrieved from the online databases of the World Bank [25]. An adjustment index (AI) was calculated using the following formula: AI = [total number of publications for the country/GDP per capita of the country]*1,000. Where GDP per capita = GDP/population of the country [5].

## Results

The total number of documents which were published in toxicology journals obtained by entering the word “toxicology” and related terms in the Scopus search engine as a source title without specifying the name of any country was 74,468 documents. This number represents the total global research productivity in toxicological journals during the past decade. Using the methodology stated above, only 1,240 (1.66% from the total global research productivity in toxicology journals) documents from 13 MEA countries were retrieved, comprising 1,158 (93.4%) original journal articles; 33 (2.7%) review articles; 27 (2.2%) meeting/conference abstracts; 12 (1.0%) letters; and 10 (0.8%) other types of publications, with an average of 124 documents per year. According to Scopus, the 13 MEA countries produced 60,477 biomedical publications during 2003–2012, which is around 49 times higher than those produced in toxicology journals.

Table 1 shows the annual number of documents published in the past decade (2003–2012). The results indicate that publications in toxicology journals during the past decade were low in the first few years but showed an obvious doubling after 2009. The quantity of publications has increased by around three-fold from 2003 to 2012.

**Table 1:** Annual number of toxicology-based publications in 13 Middle Eastern Arab countries

**Year**	**Total**
	**n = 1240 (%)**
2003	83 (6.7)
2004	85 (6.9)
2005	56 (4.5)
2006	81 (6.5)
2007	91 (7.3)
2008	101 (8.1)
2009	139 (11.2)
2010	193 (15.6)
2011	197 (15.9)
2012	214 (17.3)

The retrieved documents were published in 73 international peer-reviewed toxicology journals out of 102 peer-reviewed toxicology journals registered in Scopus (Table 2); 143 articles (11.5%) were published in *Food and Chemical Toxicology* whereas 72 (5.8%) were published in the *Journal of Environmental Science and Health, Part A: Toxic Hazardous Substances and Environmental Engineering*, and 71 (5.7%) were published in the *Bulletin of Environmental Contamination and Toxicology* journal. This was followed by 63 (5.1%) published in *Toxicological and Environmental Chemistry*, 46 (3.7%) published in *Toxicon*, 45 (3.6%) published in *Toxicology*, and 41 (3.3%) published in *Ecotoxicology and Environmental Safety*. Of the 73 journal titles, 52 (69.9%) have their IF listed in the JCR 2012; 198 documents (16.0%) were published in journals that had no official IF (Table 2). Only 21documents were published in the three journals with an IF >5.

**Table 2:** List of journals in which the 1,240 documents were published with their corresponding impact factors

**Journal**	**Frequency**	IF (2012)*
Food and Chemical Toxicology	143	3.01
Journal of Environmental Science and Health, Part A: Toxic Hazardous Substances and Environmental Engineering	72	1.252
Bulletin of Environmental Contamination and Toxicology	71	1.105
Toxicological and Environmental Chemistry	63	NA
Toxicon	46	2.924
Toxicology	45	4.017
Ecotoxicology and Environmental Safety	41	2.203
Journal of Applied Toxicology	37	2.597
Mutation Research Genetic Toxicology and Environmental Mutagenesis	37	2.22
Toxicology and Industrial Health	37	1.555
Environmental Toxicology and Pharmacology	36	2.005
Basic and Clinical Pharmacology and Toxicology	34	2.124
Human and Experimental Toxicology	33	1.453
Journal of Biochemical and Molecular Toxicology	30	1.596
Comparative Biochemistry and Physiology C: Toxicology and Pharmacology	28	2.707
American Journal of Pharmacology and Toxicology	28	NA
Toxicology and Applied Pharmacology	27	3.975
Toxicology Letters	24	3.145
Research Journal of Environmental Toxicology	22	NA
Archives of Toxicology	19	5.215
Neurotoxicology	19	2.652
Toxicology In Vitro	19	2.65
Archives of Environmental Contamination and Toxicology	17	2.012
Drug and Chemical Toxicology	16	1.293
Journal of Toxicological Sciences	15	1.380
Journal of Pharmacology and Toxicology	15	NA
Chemical Research in Toxicology	14	3.667
Toxicology Mechanisms and Methods	13	1.367
Inhalation Toxicology	12	1.894
Journal of Venomous Animals and Toxins Including Tropical Diseases	12	0.545
Aquatic Toxicology	11	3.73
Reproductive Toxicology	11	3.141
Practice Periodical of Hazardous Toxic and Radioactive Waste Management	11	NA
Toxicological Sciences	10	4.328
Clinical Toxicology	10	2.592
Indian Journal of Forensic Medicine and Toxicology	10	NA
Environmental Toxicology and Chemistry	9	2.618
International Journal of Toxicology	9	1.346
Environmental Toxicology	8	2.708
Journal of Toxicology and Environmental Health Part A: Current Issues	8	1.733
Journal of Toxicology	8	NA
Journal of Pharmacological and Toxicological Methods	7	2.150
Journal of Analytical Toxicology	7	2.107
Birth Defects Research Part B Developmental and Reproductive Toxicology	7	1.971
Journal of Occupational Medicine and Toxicology	6	1.403
Cutaneous and Ocular Toxicology	6	1.044
Forensic Toxicology	5	3.194
Journal of Environmental Pathology Toxicology and Oncology	5	0.919
Toxicological Research	5	NA
Toxicology International	5	NA
Ecotoxicology	4	2.773
Cell Biology and Toxicology	4	2.338
Regulatory Toxicology and Pharmacology	4	2.132
Toxins	4	2.129
Internet Journal of Toxicology	4	NA
Journal of Medical Toxicology	4	NA
Veterinary and Human Toxicology	4	NA
Journal of Environmental Science and Health Part C: Environmental Carcinogenesis and Ecotoxicology Reviews	3	3.565
Neurotoxicology and Teratology	3	3.181
Interdisciplinary Toxicology	3	1.346
Journal of Forensic Medicine and Toxicology	3	NA
Expert Opinion on Drug Metabolism and Toxicology	2	2.944
Cardiovascular Toxicology	2	2.351
International Journal of Medical Toxicology and Legal Medicine	2	NA
Journal of Hazardous Toxic and Radioactive Waste	2	NA
Toxicology and Environmental Health Sciences	2	NA
Particle and Fibre Toxicology	1	9.178
Nanotoxicology	1	7.844
Reviews of Environmental Contamination and Toxicology	1	4.125
International Journal of Toxicological and Pharmacological Research	1	NA
Research Communications in Pharmacology and Toxicology	1	NA
Research Journal of Toxins	1	NA
Therapeutics Pharmacology and Clinical Toxicology	1	NA

NA, Not available; IF, Impact factor.

*The IF was reported according to Institute for Scientific Information journal citation reports 2012.

When the data were analysed by country, the highest number of publications in toxicology journals was from Egypt (49.8%), followed by KSA (26.3), UAE (11.5%), and Jordan (4.8%) (Table 3). After adjusting for economy and population power, Egypt (193.6), Palestine (18.1), KSA (13.0), and Jordan (11.5) had the highest research productivity. Countries with large economies, such as the Kuwait, UAE, and Oman tended to rank relatively low after the adjustment of GDP (Table 3). The total number of citations, at the time of data analysis (August 4, 2013), was 10,991, with a mean ± SD of 8.8 ± 15.0 and median (interquartile range) of 4 (1–11). The highest median (interquartile range) number of citations was 9 (1.8–21) for Lebanon, followed by 7.5 (3–16) for UAE, 7 (3–14) for Oman. The lowest median (interquartile range) number of citations was 1 (0.0–3.8) for Iraq and 1 (0.0–5.5) for Qatar. Furthermore, the highest median (interquartile range) IF was 2.8 (2.1–3.0) for Lebanon, followed by 2.7 (1.3–3.1) for Oman, and the lowest median (interquartile range) IF was 1 (0.0–2.0) for Bahrain.

**Table 3:** Bibliometric analysis of the 1,240 documents by country

**Country**	**Number of documents**	**Total citations**	**Median (Q1–Q3) citation**	**Total IF**	**Median (Q1–Q3) IF**	**H index**	**Number (%)**^**b**^** of documents indexed in ISI**	**Number (%)**^**c **^**of documents with international authors**	**Adjustment index**^**d**^
	**n =1,240**^**a**^** (%)**^**a**^								
Egypt	617 (49.8)	5702	4 (1–11)	1277	2.2 (1.1–3.0)	33	553 (89.6)	247 (40.0)	193.6
KSA	326 (26.3)	2098	3 (1–7.5)	631.6	2.0 (1.1–3.0)	21	291 (89.3)	179 (54.9)	13.0
UAE	142 (11.5)	1691	7.5 (3–16)	324.9	2.6 (1.4–3.0)	21	128 (90.1)	94 (66.2)	3.8
Jordan	57 (4.6)	217	2 (0–6)	76.7	1.3 (0.0–2.1)	8	46 (80.7)	33 (57.9)	11.5
Kuwait	60 (4.8)	471	4 (1.3–11)	100.5	1.4 (1.3–2.2)	13	56 (93.3)	26 (43.3)	1.2
Lebanon	46 (3.7)	893	9 (1.8–21)	121.7	2.8 (2.1–3.0)	14	43 (93.5)	25 (54.3)	4.7
Oman	34 (2.9)	538	7 (3–14)	82.8	2.7 (1.3–3.1)	11	30 (88.2)	25 (73.5)	1.6
Iraq	20 (1.6)	54	1 (0.0–3.8)	24.6	1.2 (0.0–1.8)	4	13 (65.0)	10 (50.0)	3.1
Palestine	18 (1.5)	128	3.5 (1.8–10.3)	33.1	2.1 (1.4–2.2)	6	17 (94.4)	11 (61.1)	18.1
Qatar	13 (1.0)	49	1 (0.0–5.5)	17.4	1.1 (0.0–2.8)	4	7 (53.8)	9 (69.2)	0.2
SAR	10 (0.8)	35	4 (1.5–5.3)	22.2	2.6 (1.7–3.0)	4	9 (90.0)	9 (90.0)	3.0
Yemen	9 (0.7)	66	5 (1.5–10)	15.2	2.0 (0.0–2.8)	5	7 (77.8)	9 (100)	6.0
Bahrain	4 (0.3)	7	2 (0.0–2.7)	4.25	1.0 (0.0–2.0)	2	3 (75.0)	1 (25)	0.2

IF, Impact factor; ISI,Institute for Scientific Information; UAE, United Arab Emirates; KSA, Kingdom of Saudi Arabia; SAR, Syrian Arab Republic; Q1–Q3, Lower quartile – upper quartile.

^a^Total exceeds 100% because data are overlapping due to multiple collaborations.

^b^Percentage of documents indexed in ISI from the total number of documents for each country.

^c^Percentage of documents with international authors from the total number of documents for each country.

^d^An AI was calculated using the following formula: AI = [total number of publications for the country/GDP per capita of the country]*1,000. Where: GDP per capita = GDP/population of the country.

Of the 1,240 documents considered for the *h*-index, 39 had been cited at least 39 times at the time of data analysis (August 4, 2013). The highest *h*-index was 33 for Egypt, followed by 21 for KSA, 21 for the UAE, and the lowest *h*-index was 2 for Bahrain. The highest percentage of documents indexed in ISI from the total number of documents for each country was 99.4% for Palestine, followed by 93.5% for Lebanon, 93.3% for Kuwait, and the lowest was 53.8% for Qatar. Furthermore, regarding the countries that have the highest collaborations with international authors, all the documents from Yemen were published with international collaboration followed by documents from SAR (9/10, 90.0%).

The study identified 471 (38%) documents with 56 countries in MEA-foreign country collaborations. MEA actively collaborated with authors from the United States of America (n = 129, the highest number recorded), followed by the United Kingdom (n = 48), India (n = 48), Germany (n = 41), Canada (n = 36), France (n = 33), Japan (n = 29), China (n = 26), and Malaysia (n = 22) (Table 4). By region, MEA collaborated most with countries in Europe (14.4%), especially the United Kingdom and Germany (Table 4). Table 5 presents the areas of interest of the scientific articles. Pharmacology and pharmaceutics was the most researched topic, represented by 1,006 (81.1%) articles. The second most researched topic was environmental science 699 (56.4%) followed by agricultural and biological sciences 167 (13.5%) articles. On the other hand, topics such as chemistry or epidemiology and social sciences ranked low in their contribution to research output. Furthermore, Table 6 shows the first prolific toxicology authors from the 13 MEA countries with their affiliations and publication patterns. Table 7 presents a list of the 20 most cited articles originating from the 13 MEA countries.

**Table 4:** Collaborations between the 13 Middle Eastern Arab countries and foreign countries in toxicological publications

**Collaborating countries**^**a**^	**No. of documents**	**Collaborating countries a**	**No. of documents**
**MEA-MEA**	**116 (9.4%)**	**MEA-Asia-Pacific**	137 (11.0%)*
Egypt	84	India	48
Saudi Arabia	79	Japan	29
United Arab Emirates	26	China	26
Kuwait	5	South Korea	15
Jordan	13	Pakistan	10
Lebanon	2	Taiwan	4
Oman	17	Australia	4
Qatar	9	New Zealand	3
Syrian Arab Republic	3	Bangladesh	2
Yemen	2	Hong Kong	2
Bahrain	1	New Caledonia	1
**MEA-other Middle East, Africa**	55 (4.4%)*	French Polynesia	1
Turkey	14	**MEA-Europe**	178 (14.4%)*
Iran	9	United Kingdom	48
Tunisia	8	Germany	41
Libyan Arab Jamahiriya	5	France	33
Sudan	5	Czech Republic	13
Ethiopia	4	Belgium	10
South Africa	3	Austria	9
Zimbabwe	3	Netherlands	8
Israel	2	Denmark	8
Algeria	2	Italy	8
Ghana	2	Spain	6
Guinea	1	Ireland	5
Nigeria	1	Sweden	5
Zambia	1	Finland	4
**MEA-Americas**	150 (12.1%)*	Switzerland	4
United States	129	Hungary	3
Canada	36	Bulgaria	1
Puerto Rico	2	Serbia	1
Brazil	1	Slovakia	1
**MEA-Southeast Asia**	25 (2.0%)*	Bosnia and Herzegovina	1
Malaysia	22	Romania	1
Thailand	2	Albania	1
Singapore	1	Poland	1
		Croatia	1

MEA, Middle Eastern Arab countries.

^a^The study identified 471 (38%) documents with 56 countries in MEA-foreign country collaborations.

*Total exceeds 38% as data are overlapping due to multi-country collaboration.

**Table 5:** Areas of interest for published papers by the 13 Middle Eastern Arab countries

**Areas of interest**	n (%)*
Pharmacology and Pharmaceutics	1006 (81.1)
Environmental Science	699 (56.4)
Agricultural and Biological Sciences	167 (13.5)
Medicine	165 (13.3)
Biochemistry, Genetics and Molecular Biology	115 (9.3)
Chemical Engineering	30 (2.4)
Neuroscience	22 (1.8)
Chemistry	17 (1.4)
Social Sciences and Epidemiology	15 (1.2)
Planetary Sciences	13 (1.1)
Immunology	12 (1.1)
Veterinary	4 (0.3)

*Total exceeds 100% as data are overlapping due to multidiscipline interaction.

**Table 6:** The first prolific toxicology authors from the 13 Middle Eastern Arab countries with their affiliations and publication patterns

**Country**	**Total publications for the country**	**Author**	**No. (%)**^**a **^**of toxicology publications**	**Total publications for author**^**b**^	**Affiliation**
Egypt	617	Yousef, M.I.	29 (4.7)	41	Alexandria University, Department of Environmental Studies, Alexandria
KSA	326	Gondal, M.A.	25 (7.7)	124	King Fahd University of Petroleum and Minerals, Department of Physics, Dharan, Saudi Arabia
UAE	142	Petroianu, G.A.	26 (18.3)	112	Faculty of Medicine and Health Sciences, United Arab Emirates University, Department of Pharmacology and Therapeutics, United Arab Emirates
Jordan	57	Maslat, A.O.	8 (14.0)	8	Yarmouk University, Department of Biological Sciences, Irbid, Jordan
Kuwait	60	Narayana, K.	7 (11.7)	47	Health Sciences Center Kuwait Faculty of Medicine, Department of Anatomy, Safat, Kuwait
Lebanon	46	Shihadeh, A.	12 (26.1)	32	American University of Beirut, Department of Mechanical Engineering, Beirut, Lebanon
Oman	34	Ali, B.H.	17 (50.0)	58	Sultan Qaboos University, Department of Pharmacology, Muscat, Oman
Iraq	20	Mohammad, F.K.	5 (25.0)	26	University of Mosul, Department of Physiology, Biochemistry and Pharmacology, Mosul, Iraq
Palestine	18	Zyoud, S.H.	6 (33.3)	49	Poison Control and Drug Information Center (PCDIC), College of Medicine and Health Sciences, An-Najah National University, Nablus, Palestine
Qatar	13	Busselberg, D.	3 (23.1)	3	Weill Cornell Medical College in Qatar, Doha, Qatar
SAR	10	Ahmed, S.	4 (40.0)	21	International Center for Agricultural Research in the Dry Areas Syria, Aleppo, Syrian Arab Republic
Yemen	9	Al-Zubairi, A.S.	2 (22.2)	29	Sana’a University, Faculty of Medicine, Sana’a, Yemen
Bahrain	4	Sequeira, R.P.	1 (25.0)	42	Arabian Gulf University, Department of Pharmacology and Therapeutics, Manama, Bahrain

UAE, United Arab Emirates; KSA, Kingdom of Saudi Arabia; SAR, Syrian Arab Republic.

^a^Percentage of toxicology publications for the first prolific toxicology author from the total number of documents for each country.

^b^Total of all publications for each author during the period of study.

**Table 7:** The top 20 cited toxicology articles from the 13 Middle Eastern Arab countries in Scopus

**SCR**^**a**^	**Authors and year of publication**	**Title**	**Journal name**	**Times cited**^**b**^
1^st^	Ali et al., 2008	Some phytochemical, pharmacological and toxicological properties of ginger (*Zingiber officinale* Roscoe): A review of recent research	*Food and Chemical Toxicology*	191
2^nd^	El-Demerdash et al., 2004	Cadmium-induced changes in lipid peroxidation, blood hematology, biochemical parameters and semen quality of male rats: protective role of vitamin E and β-carotene	*Food and Chemical Toxicology*	175
3^rd^	Shihadeh, 2003	Investigation of mainstream smoke aerosol of the argileh water pipe	*Food and Chemical Toxicology*	162
4^th^	El-Demerdash, 2005	Biochemical study on the hypoglycemic effects of onion and garlic in alloxan-induced diabetic rats	*Food and Chemical Toxicology*	153
5^th^	Shihadeh and Saleh, 2005	Polycyclic aromatic hydrocarbons, carbon monoxide, ‘tar’, and nicotine in the mainstream smoke aerosol of the narghile water pipe	*Food and Chemical Toxicology*	147
6^th^	Jurjus et al., 2004	Animal models of inflammatory bowel disease	*Journal of Pharmacological and Toxicological Methods*	124
7^th^	Badary et al., 2003	Thymoquinone is a potent superoxide anion scavenger	*Drug and Chemical Toxicology*	101
7^th^	Mohamed et al., 2003	Estimation of microcystins in the freshwater fish *Oreochromis niloticus* in an Egyptian fish farm containing a Microcystis bloom	*Environmental Toxicology*	101
9^th^	Ali and Al Moundhri, 2006	Agents ameliorating or augmenting the nephrotoxicity of cisplatin and other platinum compounds: a review of some recent research	*Food and Chemical Toxicology*	98
10^th^	Radwan and Salama, 2006	Market basket survey for some heavy metals in Egyptian fruits and vegetables	*Food and Chemical Toxicology*	82
11^th^	Ali, 2003	Agents ameliorating or augmenting experimental gentamicin nephrotoxicity: some recent research	*Food and Chemical Toxicology*	79
12^th^	Yousef, 2004	Aluminium-induced changes in hemato-biochemical parameters, lipid peroxidation and enzyme activities of male rabbits: protective role of ascorbic acid	*Toxicology*	77
13^th^	Yousef et al., 2006	Deltamethrin-induced oxidative damage and biochemical alterations in rat and its attenuation by Vitamin E	*Toxicology*	72
14^th^	Shalan et al., 2005	Amelioration of lead toxicity on rat liver with Vitamin C and silymarin supplements	*Toxicology*	71
15^th^	Abdel-Wahhab and Aly, 2005	Antioxidant property of *Nigella sativa* (black cumin) and *Syzygium aromaticum* (clove) in rats during aflatoxicosis	*Journal of Applied Toxicology*	69
16^th^	El-Sharaky et al., 2007	Protective role of selenium against renal toxicity induced by cadmium in rats	*Toxicology*	67
17^th^	Mansour, 2004	Pesticide exposure – Egyptian scene	*Toxicology*	61
17^th^	Yousef et al., 2003	Changes in some hematological and biochemical indices of rabbits induced by isoflavones and cypermethrin	*Toxicology*	61
19^th^	Raza and John, 2006	4-Hydroxynonenal induces mitochondrial oxidative stress, apoptosis and expression of glutathione S-transferase A4-4 and cytochrome P450 2E1 in PC12 cells	*Toxicology and Applied Pharmacology*	60
20^th^	Sepetdjian et al., 2008	Measurement of 16 polycyclic aromatic hydrocarbons in narghile waterpipe tobacco smoke	*Food and Chemical Toxicology*	57

SCR, Standard Competition Ranking.

^a^Equal articles have the same ranking number, and then a gap is left in the ranking numbers.

^b^Time cited at the time of data analysis (December 13, 2013).

## Discussion

This study was limited to 1,240 documents extracted from Scopus, bearing MEA countries affiliation addresses and, therefore, cannot be generalised to the toxicological literature covered by other databases such as Google Scholar. However, the study does give a clear picture about the characteristics of the documents from MEA countries published in foreign indices, especially those indexed by Scopus. Although the number of citations for each publication might differ from one search engine to another, the Scopus search engine remains one of the best available tools for analysing and tracking citations, and comparing citations among different research groups and different institutions [26]. A study that compared PubMed, Scopus, Web of Knowledge, and Google Scholar has found that PubMed remains an important resource for clinicians and researchers, while Scopus covers a wider journal range and offers the capability for citation analysis [4,26-28].

In the present study, bibliometric indicators were used to describe scientific activity in the field of toxicology in 13 MEA countries during the last decade. Based on the authors’ knowledge, this is the first article to analyse the quantity and quality of toxicology-based research from the Arab world. Research indicators showed that research activity in this field was neglected in most MEA countries. The total publications found in Scopus between 2003 and 2012 showed a yearly increase. Most countries experienced increases in the absolute number of documents produced in the field of toxicology over time. Furthermore, the current study showed low research output in some countries. In our study, we compared the toxicological research performance in the MEA countries with that in non-MEA countries. The study shows that MEA countries are lagging behind in the number of toxicological research publications. Furthermore, they are also lagging behind when the number of toxicological publications in the MEA countries is compared with other biomedical research publications in the same region. Toxicology productivity has followed the general explosion in scientific productivity observed in the last decade and especially in recent years [5,11,13] as well as following the biomedical research performance in the Arab world in the last decades [5,29,30].

Bibliometric descriptors for documents published by toxicologists and in toxicology journals are presented in Tables 3 and 5. As can be seen, each country’s behaviour was different. Our study showed that there were some countries, such as Egypt and KSA, where their total toxicology productivity during this 10-year period was clearly higher than in the remaining countries. Previous studies reported that Egypt and KSA had the most biomedical publications among the Arab countries [29,31]. Our results are similar, as these two countries had the highest number of toxicological research publications in the Arab world. In our study, the ranking of countries after adjusting for economy and population parameters differed conspicuously from those based on absolute production. After adjusting for economy and population power, Egypt, Palestine, KSA, and Jordan had the highest research productivity. We did not find any study similar to ours, thus we are unable to discuss this point in light of other results. However, some studies using the same tool for analysis have reported similar findings [5,10,29,30,32,33]. Countries with rapidly growing economies, which results in more funding and investments for research, contribute to the tendency of increasing number of toxicology publications such as KSA. Based on our findings, besides GDP, population size is one of the main factors related to research productivity, as was observed for Egypt; this activity depended on population size, socio-economic status, or overall scientific activity of the country [11]. The annual number of documents published indicates that research productivity in toxicology journals during the past decade was low. Several studies have discussed the reasons leading to the scarcity of medical research in most Arab regions [5,10,29,30]. These studies suggested that the regional conflict has been considered a main cause for the paucity of medical publications in some Arab countries. Furthermore, a lack of funding, freedom, and democracy may contribute to low scientific research output in the Arab world [10,29,30]. All these reasons have to be taken into consideration if the governments in the Arab regions wish to develop the status of their scientific research output.

In our study, the average citation rate for publications from MEA countries was 8.8 citations per article. This finding was consistent with general average citations of toxicological journals [15,34]. Furthermore, this is slightly less than the average citation rate for most journals in other scientific disciplines [15,34]. Overall, toxicology journals as a group have low citation numbers compared to other scientific disciplines. This is likely attributed to several facts. First, the number of researchers of toxicology is small, leading to relatively fewer publications being published in peer-reviewed toxicology journals compared with other disciplines. Second, the apparent narrow focus of toxicology journals may encourage researchers who have some connection to the field of toxicology to publish their results in journals that may have a larger audience than that of toxicology journals [15,35]. This exact scenario was demonstrated in the emergency medicine literature by Callaham et al. [36]; they found that publications by emergency medicine researchers were cited more than two times as often when published in non-emergency medicine journals.

The results of this study showed that the most cited articles published from MEA countries were mainly those in the field of public health. It was very striking that three articles from the top 20 cited articles published from MEA countries were articles related to tobacco smoking. Smoking is considered a major risk factor for several major diseases including heart disease, cancer, and chronic obstructive pulmonary disease, and is also the most preventable cause of morbidity and mortality contributing to around half a million deaths every month, a condition that is definitely to worsen in the future [37]. Tobacco smoking is on rise and as a multi-disciplinary field of study; it has resulted in growing research that takes into account almost all those regions that have experienced the greatest increases in bioscience and healthcare science production.

In the current study, topics such as epidemiology and social sciences ranked low in their contribution to research output in the field of toxicology. One of the scientific challenges of toxicology in the Asian region is the limited availability of epidemiological information. This field of study is neglected in most countries. Unfortunately, most of the available epidemiological data in this region have been published in different formats, and some documents were extracted from files of poisoned patients while others from poison centres, in which the outcome and frequency of poisonings were different. Thus, a clear picture of poisoning epidemiology is necessary in this region [38]. Another possible explanation for this is the lack of peer reviewed local and regional journals that are indexed in Scopus. In 2012, there were 102 journals indexed under “toxicology”, but none of them belonged to MEA countries. Thus, MEA countries are recommended to establish peer-reviewed journals to promote science in middle- and low-income countries, which could be submitted for indexing in Scopus. Publication in Asia-specific or regional journals would hopefully serve as another milestone for toxicologists in the region and will promote research about epidemiological interests that have low priorities in higher-income countries’ journals [39].

While case reports are often the most read content of journals, and they significantly advance our understanding of a particular clinical toxicology syndrome, treatment, mechanism, or are supportive of a new hypothesis [34] or serve as early warning signals for the adverse effects of new drugs and unique clinical presentations from common drugs [40], they were ranked low in their contribution to research output in the field of toxicology from MEA countries. One possible explanation for this is the low number of publications from MEA countries which were published in clinical or human toxicology journals. The results of our study showed that *Food and Chemical Toxicology*, *Journal of Environmental Science and Health, Part A: Toxic Hazardous Substances and Environmental Engineering*, *Bulletin of Environmental Contamination and Toxicology* journal, *Toxicological and Environmental Chemistry*, *Toxicon*, *Toxicology*, and *Ecotoxicology and Environmental Safety* have the largest share from MEA countries’ publications. These journals were more interested in areas of environmental science, agricultural and biological sciences, biochemistry, genetics and molecular biology, and chemical engineering rather than clinical cases.

It is noteworthy that some MEA authors have succeeded in publishing in high-quality journals like *Particle and Fibre Toxicology*, *Archives of Toxicology*, *Toxicological Sciences*, *Nanotoxicology*, and *Toxicology*. International collaborations might help MEA researchers to publish in journals with a high IF. Comparing toxicology to areas such as molecular biology and genetics, where new discoveries are made almost every day, toxicology is a more slowly advancing science. This can result in wide disparities between the citations of journals in different fields in comparison to a journal with a narrow field [34]. This may be one reason why journals like *The Lancet*, *JAMA*, *Nature*, *Science*, and the *New England Journal of Medicine*, whose content encompasses the entire scope of medicine and science, are always among the journals with the highest citations, which in turn leads to high IFs. Since toxicology is a very narrow field with a very small and specialized readership, it should not be astonishing that toxicology journals have small numbers of citations, which leads to average IFs [15,41]. Therefore, it would have been more interesting to know how the growth of toxicology in these countries was in quality rather than in quantity, as shown by the low median citations and IFs. The preparation of quality research documents requires significant effort and time. Publishing in high-impact journals allows established researchers to be able to obtain further funding to support collaborative research and for young researchers to be more competitive in career advancement [42].

Moreover, MEA authors mainly collaborated with authors from the United States of America, India, United Kingdom, Germany, Canada, China, France, Japan, and Malaysia. This may be because most MEA academics graduated from or were trained in these countries. Investigators who are open to collaborations and those who seem to adequately manage their collaborations produce a superior product that results in a higher impact and higher citation rates [43]. The factors in favour of increasing collaborations internationally cannot be ignored; these are the results of easier access to public financing, opportunities to attain higher productivity, and aspirations for greater prestige and visibility resulting from collaboration with renowned research groups [44,45]. In addition to these advantages of collaboration, follow-up research expertise of other countries, developed or developing, is another key factor for facilitating applicable and translatable research in countries that historically lack it.

This study shows the first prolific toxicology authors from the 13 MEA countries with their affiliations and publication patterns, indicating their active roles as writers. In most universities worldwide, promotional criteria require academics to show their active involvement in research as reflected by the ranking as first prolific toxicology authors. Often the Deanship of Scientific Research will be asked by university administrators to provide such evidence and the analysis of the names of productive authors becomes necessary. The improvement of some institutes may be attributed to the emphasis by universities for academics to publish in journals indexed by the ISI databases and Scopus. Information about trends and productivity reveals the intellectual output of toxicology works published in Scopus and is useful to university administrators when evaluating the yearly performance of university faculties in the light of university rankings among Arab universities.

In most MEA countries, the underdevelopment of toxicology is primarily due to improper educational policies. This region still lacks well-defined and elaborate postgraduate toxicology programmes at the university level, and there is a shortage of human resources in this field. Therefore, the formulation of a life sciences curriculum that comprises basic and applied toxicology and environmental sciences-related subject matter is needed. Recently, most institutes of education in MEA countries have undertaken a few important steps in this direction. Young researchers in MEA countries understand the principles of toxicology only at higher degree levels, that is, doctoral degree or postgraduate programmes. Therefore, the sharing of relevant research questions by developed and developing countries can lead to research opportunities [46]. Lastly, developing a culture of research in MEA countries, led by a researcher with an expertise in biostatistics and epidemiology, and who may be based in academia, is another necessary key to having a sustained productive research effort [46].

This study is not without limitations, most of which are the same as those of studies performed in other biomedical fields. First of all, we used Scopus criteria for including toxicology journals in our study. Articles published in non-Scopus-cited journals were not included; however, they might contribute to scientific productivity. In addition, we searched only for journals included in the “Toxicology”-related term of SCOPUS, although many articles in the toxicology field are published in other toxicology journals, with a wider field of interest, such as medicine and pharmacology. Furthermore, some conference abstracts may be published by certain journals which may be then published in the same or different journals in different year as original journal articles. Another limitation is that some international journals do not recognise countries like Palestine as a separate country and publications from Palestine may be affiliated with Israel as a country. Therefore, some publications from Palestine might be missed from our analysis.

## Conclusions

This study, to our knowledge, is the first detailed analysis of the research publication output in the field of toxicology of an institution setting in MEA countries. This paper’s main goal is to direct attention and to open the doors for a scientific discussion among toxicology professionals and academics. The present data show a promising rise and a good start for toxicology research activity in toxicology journals from the Arab world. Research output is low in some countries, which can be improved by investing in more international and national collaborative research projects in the field of Toxicology. Epidemiology of poisoning or case reports in toxicology are neglected in the MEA region. Most toxicology aspects, such as epidemiological data, are suitable for regional journals rather than international journals. Thus, MEA countries are recommended to establish peer-reviewed journals to promote science in middle- and low-income countries that could be submitted for indexing in Scopus.

## Abbreviations

AI: Adjustment index; GDP: Gross domestic product; IF: Impact factor; IRB: Institutional review board; ISI: Institute for Scientific Information; JCR: Journal citation reports; KSA: Kingdom of Saudi Arabia; MEA: Middle Eastern Arab; SAR: Syrian Arab Republic; UAE: United Arab Emirates.

## Competing interests

The authors declare that they have no competing interests.

## Authors’ contributions

All authors were involved in drafting the article, and all authors approved the final version to be submitted for publication. SZ conceived of the study and design, organized and supervised the data collection, and provided analysis, interpretation, and writing. SA and WS participated in the study design, and provided critical revision of manuscript for important intellectual content. RA was involved in concept and editing the manuscript.
